# Speaking of survival: oncologists’ approaches to risk behavior conversations with cancer survivors

**DOI:** 10.1007/s11764-025-01798-1

**Published:** 2025-04-15

**Authors:** Savanna Kerstiens, Courtney Lynam Scherr, Maia Jacobs, Allison J. Carroll, Monisola Jayeoba, Elyse Renee Daly, Siobhan M. Phillips, Brian Hitsman, Sofia F. Garcia, Bonnie Spring

**Affiliations:** 1 School of Communication, Northwestern University, Evanston, IL USA; 2 Robert H. Lurie Comprehensive Cancer Center, Northwestern University, Chicago, IL USA; 3 Feinberg School of Medicine, Northwestern University, Chicago, IL USA

**Keywords:** Risk communication, Diet, Smoking, Physical activity, Clinician communication, Survivorship

## Abstract

**Purpose:**

This study explored oncologists’ reported conversations with adult cancer survivors about three modifiable risk behaviors: poor diet, smoking, and insufficient physical activity. These behaviors can increase disease recurrence and early mortality. By asking oncologists to report their approach to these discussions, we can begin to identify areas for improvement.

**Methods:**

We conducted semi-structured interviews with 17 oncology clinicians from various subspecialties. We identified themes using inductive thematic analysis.

**Results:**

Four major themes were identified. Participants reported only sometimes explicitly discussing risk behaviors and avoiding sensitive topics like weight. They described tailoring their conversations around what they believe a patient can handle to maintain a positive relationship or avoid hurting patients’ self-image. Participants explained that they rely on specialists or programs for specific risk management strategies and indicated that patients most often initiate conversations about lifestyle risks. Participants avoided or indirectly discussed lifestyle risks.

**Conclusion:**

The oncologists’ reported risk communication with survivors suggested a delicate balance between patient-centered communication and benevolent bias—where oncologists tailor conversations based on their perceptions of what a patient can handle. Indirect discussions may lead survivors to undervalue the importance of addressing these risks. To enhance communication effectiveness and avoid unmanaged risk behaviors, oncologists need support to be more fully engaged in specific risk management conversations with cancer survivors.

**Implications for Cancer Survivors:**

Oncologists’ benevolent bias may lead to inadequate risk management by the clinician and result in survivors underestimating the importance of specific risk factors, potentially leading to poorer health outcomes.

**Supplementary Information:**

The online version contains supplementary material available at https://doi.org/10.1007/s11764-025-01798-1.

## Introduction

Adult cancer survivors who are post-treatment with curative intent (hereafter referred to as cancer survivors) are at increased risk for cancer recurrence, secondary cancers, other diseases, and earlier mortality often due to cancer- or treatment-related comorbidities and modifiable risk behaviors like poor diet, smoking, and insufficient physical activity [1–5]. Poor diet can reduce patients’ quality of life and exacerbate risk for functional decline and comorbidity; smoking can lead to disease progression, second primaries, and increased comorbidity; and physical inactivity can influence cardiorespiratory fitness, strength, fatigue, and mortality [6]. Patients with fewer risk behaviors or who proactively maintain healthy behaviors can decrease their risk and improve health outcomes and quality of life [2, 5, 7, 8]. Helping cancer survivors manage their diet, quit smoking, and engage in physical activity is critical to mitigating the risk of cancer recurrence and adverse health outcomes.

Several organizations (e.g., American Society of Clinical Oncology [9, 10], American Cancer Society (ACS) [11, 12], National Comprehensive Cancer Network (NCCN) [13, 14]) have published guidelines for cancer survivors for maintaining a healthy diet and weight, quitting smoking, and increasing physical activity, and advise oncologists to perform risk behavior counseling. Oncologists’ risk behavior counseling can positively enhance patients’ self-efficacy and motivation and, in turn, promote behavior change promote behavior change [15] and is positively associated with cancer survivors’ adoption and maintenance of a healthy diet, smoking cessation, and appropriate levels of physical activity [5, 10, 16]. However, clinician communication is more nuanced than simply providing recommendations—what they say and how they say it can influence patient engagement in behavioral interventions [17].

Studies investigating clinician recommendations for risk management behaviors, such as cancer screening, show that the content of the conversation, such as whether clinicians provide counseling on early detection behaviors and screening, and the quality of discussions, such as clinician enthusiasm, encouragement, and thorough explanation, had an additional influence on patients’ behaviors [17, 18]. When clinicians told patients in the general population (i.e., not cancer survivors) the importance of cancer screening, its impact on future risk for disease, and its urgency, patients were more likely to engage in recommended screening and prevention than if clinicians offered low-quality recommendations framing the behavior as less important or nonurgent [17, 19]. Oncologists report assessing and advising their cancer patients’ lifestyle habits before and during treatment [20]; however, they also tend to prioritize discussions about cancer risk and medication management rather than lifestyle-related health risks [4]. Moreover, oncologists have reported having fewer lifestyle behavior conversations during the survivorship phase of care [21], often related to barriers such as perceived irrelevance to their clinical role [22], inappropriate timing, insufficient training [20, 21], and concerns about appearing insensitive or accusatory [23]. Recommending improvements to diet and physical activity and promoting smoking cessation are crucial in cancer survivorship, and the content and delivery of those recommendations likely play a significant role in uptake.

Surprisingly, little research has explored how oncologists discuss these risk behaviors with cancer survivors. Exploring how recommendations are offered and identifying areas for improvement is vital, considering few cancer survivors report receiving recommendations about risk management [1, 23, 24] or meet recommended guidelines [16, 20]. To understand more about how oncologists deliver recommendations, this study examined oncologists’ reported communication strategies for discussing diet, smoking, and physical activity with cancer survivors.

## Methods

### Study design

This qualitative study took place between August and November 2023 and investigated oncologists’ self-reported discussions with cancer survivors about diet, smoking, and physical activity during routine visits. Semi-structured interviews were conducted with oncologists providing survivorship care across 11 clinics in an academic-affiliated healthcare network to inform the development of a telehealth risk behavior program for cancer survivors [25]. This study was approved by the institution’s Institutional Review Board.

### Participants and procedures

We recruited oncologists from various specializations (e.g., breast, lung, brain, and radiation oncology) in our healthcare network to obtain diverse perspectives regarding the role of risk behaviors in cancer recurrence, treatment regimens and their side effects, and the perceived relevance of risk behaviors during survivorship [9, 26]. Clinicians were eligible if they were over 18 years old, spoke English, were not cognitively impaired, and worked in the healthcare network.

Recruitment involved a modified snowball sampling method. We emailed oncologists within the network and used participant recommendations to build the sample. Three study team members with expertise in qualitative interviewing conducted virtual interviews. All participants provided verbal consent before starting and recording the interview. Recruitment occurred until saturation was achieved, and no new information was observed in the data [27]. Participants who completed the interview received $50 in appreciation for their time.

### Data collection and analysis

Using a semi-structured interview guide (Supplemental File 1) developed by the study team, which included behavioral scientists and psychologists who work with oncology patients and survivors, we asked participants about their current approaches to discussing diet, smoking, and physical activity with their cancer survivor patients. Interviews were recorded, transcribed verbatim, and anonymized. Data were coded via inductive thematic analysis [28, 29] using MAXQDA for data management (VERBI software, 2022, Berlin, Germany) [30]. Two authors independently reviewed two transcripts and familiarized themselves with the data, noting initial ideas [29, 31]. They met to discuss initial annotations and create a preliminary codebook. Next, they independently coded an additional two transcripts, met, and discussed their initial findings and discrepancies, revising the codebook accordingly. They applied this iterative and consensus-based approach to coding the remainder of the transcripts, maintaining detailed notes of concepts to identify themes [29]. Preliminary themes were reviewed and discussed with coauthors and other colleagues. Final themes were defined and named during a recursive process, returning to original codes when needed [29, 31].

## Results

Seventeen clinicians participated in the interviews (Table 1). A slight majority was female (*n* = 9; 53%), most were White (*n* = 13; 76%), and participants were 48 years on average (range, 35–72). Participants had been practicing oncology for an average of 17 years (range, 1–40). The greatest number of participants practiced in breast oncology (*n* = 4; 24%) and neuro-oncology (*n* = 3; 18%). Four themes were identified: (1) Inconsistencies: timing and motivations for discussing patient risk behaviors; (2) Benevolence: framing biases in discussing patient risk behavior; (3) Safeguard: avoiding discussions about patient risk behavior until patients initiate; and (4) Reliance: external sources necessary when discussing patient risk behavior. Next, we discuss the participants’ reported strategies, motivations, and attitudes about discussing diet, smoking, and physical activity with their cancer survivor patients. Participants are referred to by cancer type and number of years of experience in parentheses.

**Table 1:** Participant demographics (*n* = 17)

Demographics	*n* (%)
Gender:	
Female	9 (53)
Male	7 (47)
Age (mean, (sd))	48 (10)
Race and ethnicity	
White	13 (76)
South Asian	2 (12)
Hispanic	1 (6)
Indian	1 (6)
Years of experience (mean (sd))	17 ± 12
Clinical practice characteristics sub-specialty	*n* (%)
Breast Oncology	4 (24)
Neuro-Oncology	3 (18)
Genitourinary Oncology	2 (12)
Thoracic Oncology	2 (12)
General Oncology	2 (12)
Radiation Oncology	1 (6)
Gastrointestinal Oncology	1 (6)
Hematology	1 (6)
Cancer Genetics	1 (6)

### Oncologists’ inconsistencies: timing and motivations for discussing patient risk behaviors

Participants gave a range of responses about how often they address physical inactivity, diet, and smoking risks with patients during survivorship. A few thought cancer survivors were poised to benefit from conversations about diet, physical activity, and smoking cessation and reported commonly addressing them in clinic. As one participant shared,

*It’s usually in my top 3 encounter priorities…I will usually talk to the patient first about overall how they’re feeling and anything that would trigger any concern for a cancer recurrence or progression…I always talk to them. Top 3 is about kind of diet exercise hydration. All of those things. Yeah, stop smoking. All of that.* (Breast Oncology, 10y).

Several participants articulated that oncology as a specialty is more focused on treating cancer and current quality of life than long-term risk management, indicating that survivorship was about disease surveillance, and as such, reported rarely addressing risk behavior. For example, risk behaviors are, “At the bottom of the list. So we typically don’t address those types of things. You know, we’re, you know, oncologists. So we’re busy focusing on treating the tumor” (Neuro-oncology, 17y). Most participants fell in the middle, stating they sometimes (but not always) bring up these risk factors, and not necessarily with a strong emphasis.

Similarly, the type of cancer a clinician specializes in and the patient population they serve influence their conversations regarding risk behaviors. For instance, a neuro-oncologist suggested, “So with brain cancer, what’s different about this, compared to other cancers that affect other organs is that, like a less aggressive cancer can still cause tremendous neurologic deficits so kind of staying physically active and mobile is helpful” (Neuro-Oncology, 7y). Some did not feel that long-term risk management conversations fit into their scope of practice due to their specialty. For instance, one radiologist described the limited-term relationship they have with most patients, “We don’t really do long-term longitudinal follow-up on most patients. So, coming from that perspective, addressing some of these longer-term health things is not high on our radar.” (Radiation Oncology, 23y).

*Furthermore, most participants said they are selective in the types of risk they address. For instance, I would say, it’s mostly on the physical activity, and I really have not asked them like, Are you drinking now? Are you smoking now? But those questions usually come up in the beginning when I just met them, and I usually don’t bring it up again*. (Gastrointestinal Oncology, 1y).

Participants explained that smoking was not something they brought up with cancer survivors because most believed their patients did not smoke or, if they did, they had already addressed that risk at intake. Similarly, a few participants reported they do not explicitly talk about weight, “I don’t ask so much like, ‘Have you gained weight?’ because that takes us like too much of a slippery slope that I don’t have time to address” (Breast Oncology, 18y). Participants felt that the patient’s weight and smoking were sensitive topics. There was variability in the frequency of discussions about modifiable risks, motivations for having these discussions, and the types of risks participants reported discussing with cancer survivors.

### Oncologists’ benevolence: framing biases in discussing patient risk behavior

Participants across sub-specialties commonly described framing risk behaviors as maintaining a generally healthy lifestyle. For example, “it’s more stress kind of the general health rather than like must do one certain thing, otherwise, your cancer is going to come back, right. It’s more like you should do all these things because it’s good for your health” (Breast Cancer, 18y). A couple explained that they take a more positive, proactive approach (general healthy lifestyle) versus a negative reactive approach (specific activities will prevent cancer recurrence) because they do not want cancer survivors to feel blamed for their cancer. As one participant explained, “you don’t want to hint that they cause this…You never want them to feel that guilt” (Radiation Oncology, 23y). They indicated that they frame risk behaviors as generally healthy things to do to avoid overwhelming their patients or damaging the clinician-patient relationship.

A few, most commonly those who treat breast or neurological cancers, described framing conversations about diet and exercise around mitigating the long-term effects of cancer and its treatment instead of as prevention. In some cases, cancer survivors continue treatment such as hormone therapies post-primary treatment to reduce the risk of recurrence, which have ongoing side effects that may be addressed through physical activity. For example, breast oncologists explained that they tend to provide general recommendations about activity and diet in response to surgically or medically induced menopause or weight concerns due to medications, “people, you know, who are concerned about weight gain or concerned about their bone health our message is always like everything you could do, physical activity-wise, is going to help your symptoms, it’s going to help your bone health” (Breast Oncology, 18y). As another example, a participant said they recommend physical activity when patients experience treatment fatigue from long-term treatments.

*Fatigue is kind of the major side effect that people experience from the therapies that they’re on, and that they’re on long term. And we discuss that, you know, it’s kind of a vicious cycle. So, the best way to counteract fatigue is to remain active, and once you’re active you have more energy and then you can keep being active.* (Genitourinary Oncology, 14y).

Moreover, most participants said they personalize conversations about risk management around what they believe to be an achievable goal for their patients, for example,

*With patients who are physically inactive or overweight, I talk to them about different alternative modalities, because I think a lot of times they just think they have to go to the gym, or they have to walk. So I’ll talk to them about chair yoga, Pilates, certain just kind of stretching exercises* (Breast Oncology, 10y).

Framing the conversation around achievable goals was believed to help reduce barriers to engaging in risk-reduction behaviors.

### Oncologists’ safeguard: avoiding discussions about patient risk behavior until patients initiate

A few participants acknowledged not discussing specific risks unless the patient brings them up. For instance, “I feel like it’s something that has to be so obvious, or the patient brings it up as something that they are struggling with, and that’s bothering them” (Thoracic Oncology, 38y). They reported that patients most often ask about diet, such as a ketogenic diet or “special diets for cancer patients” (General Oncology, 30y). For example, one breast oncologist said, “Everyone wants to know, like, is there a specific diet, like do I have to be vegan?” (Breast Oncology, 18y). One clinician suggested that patients ask about marijuana, CBD, or THC, which then offers “A segue into, you know, smoking, smoking in general. You know, and then other substances secondary to that” (Neuro-Oncology, 6y).

Participants indicated that patients often brought up concerns about weight, especially breast cancer survivors who were on medications known to cause weight gain, which then allowed them to discuss diet and physical activity. For example,

*I’ll tell you that the one thing that patients do bring up to me on a regular basis is their frustration with their weight. What can they do about it? So that’s actually an avenue that I use to then bring up the diet and the exercise* (Breast Oncology, 30y).

Some participants explained that talking about physical activity and diet went hand in hand; for example, if the clinician brings up physical activity, the patient might then bring up diet or weight: “I will often, you know, ask them what they’re doing for physical activity. I don’t talk as much about diet or portion size, unless, although a number of them do bring it up. But I do pretty regularly ask, well, physical activity” (Genitourinary Oncology, 14y).

Participants also reported that survivorship is when patients ask for advice about reducing their risk for cancer recurrence. They view this as an opportunity to discuss diet and exercise with motivated patients. For instance,

*So I always have my antennas up. When patients say, you know, I’ll do anything to stay cancer-free, and I’ll say I’m glad you said that, because there’s a couple of things that we could do. We could get you down to your ideal body weight; we could work on diet and exercise* (Genitourinary Oncology, 21y).

### Oncologists’ reliance: external sources necessary when discussing patient risk behavior

A few participants reported engaging patients in these discussions when patients initiated them; however, they relied on external sources to have more detailed conversations and facilitate behavior changes for more specific support. For example,

*If I have a patient express to me that they’re prepared to, you know, try to quit smoking and they maybe weren’t in the past, I’m more than happy to reach out to their primary [care provider] to help with that but in terms of like actual boots on the ground steps to make that happen I, you know, I’m not super familiar with that kind of stuff and would probably try to get the primary involved* (Hematology, 4y).

Participants said they often rely on standardized guidelines (e.g., from ACS) to provide treatment recommendations, and often refer to other resources or programs. As one breast cancer oncologist said,

*I do try to counsel patients to maintain a healthy weight as part of cancer, you know, ACS recommendations, and to try to have a goal of about 150 min of exercise per week. So I kind of tell them the guidelines and we’ll refer them to like our living well program which is like supportive oncology… But yeah, kind of beyond that, we don’t necessarily follow the patients in these efforts. I’m kind of just like letting them know about you know about this program, and then just kind of leaving it at that* (Breast Oncology, 11y).

Many said they refer patients to specific programs to help assist with risk management, “…if people have lost weight, how they can try to keep the excess weight off, or, you know, avoid regaining the lost weight… since a lot of people will have specific diet questions, I’ll usually refer them to a dietitian” (General Oncology, 40y). Likewise, for more specific guidance and follow-up, participants believed the patient’s primary care clinician should manage risk behaviors; as one participant said, “I think the ideal with survivorship is that plan is sort of passed on to the primary care physician” (Thoracic Oncology, 38y).

Some participants indicated they lack the training or knowledge to give patients specific recommendations and thus rely on external resources: “I don’t think I have personally enough training or knowledge to know to give them specific recommendation on what they should do” (Gastrointestinal Oncology, 1y). Likewise, one general oncologist said they rely on external resources for particularly motivated patients:

*It is important. For sure. But most of the time, it’s a lower priority, and we don’t get to it during the encounter… So, I don’t usually have to employ any strategies. I get other people involved for motivated patients and try to get the patient motivated and interact with those folks* (Genitourinary Oncology, 21y).

All participants recognized the importance of external resources for assisting patients in managing their risk behaviors due to barriers such as lack of time, lack of training, or viewing it as a lower priority.

## Discussion

This study examined oncologists’ reported discussions about poor diet, smoking, and physical inactivity with survivor patients. Our findings confirm those of prior studies showing oncologists view risk management and lifestyle advice as beyond their professional scope or expertise and are, therefore, unlikely to provide specific behavior change guidance and recommendations to cancer survivors [5]. Also consistent with prior research, participants described framing their discussions about risk behaviors as providing general health benefits and managing late and long-term treatment effects rather than preventing cancer recurrence [22]. Unique to this study, we identified a potential bias that may influence oncologists’ decisions to discuss risk behaviors with cancer survivors.

Participants’ descriptions of their approach to risk conversations during survivorship align with patient-centered communication—a healthcare approach that prioritizes patients’ values, preferences, and needs [32]. Participants explained that their subspecialty and cancer type, their patient population’s well-being, and the lasting treatment effects largely influenced their conversations about cancer risk management during survivorship. For example, regarding physical activity, neuro-oncologists focused more on general well-being, breast cancer specialists emphasized reducing side effects from long-term treatments, and gastrointestinal oncologists addressed acute survivorship issues while referring patients for specialized assistance. Despite these differences, all oncologists shared a common approach to discussing risk behaviors: they remain general in their advice, fearing more specific guidance might overwhelm patients, damage the clinician-patient relationship, or make patients feel blamed for their cancer. Their discussions around lifestyle risk behavior management depended on what they believed to be achievable goals for their patients. This tendency to decide whether or how they talk about risks based on their perceptions about what the patient can handle has been identified in prior literature as “benevolent bias” [33]. Benevolent bias is the decision of the clinician to avoid certain topics they believe may be overwhelming for the patient based on their interpretation of the patients’ context.

Benevolent bias is consistent with facework strategies frequently used by healthcare professionals when conveying bad news to patients [34]. Facework encompasses communication strategies used to maintain and protect one’s own or others’ self-image during social interactions [35]. In the case of survivorship, oncologists may perceive discussions of a patient’s risk behaviors as face-threatening to the patient, resulting in communication that consists of them being less assertive than warranted [34]. This approach may not be ideal for all patients.

Even when well-intentioned, clinicians’ preconceptions about the patient’s status and abilities may hinder positive outcomes if it does not align with the patient’s needs or preferences [32, 36]. For example, clinicians tailoring the conversation around risk in general terms for a patient who prefers clinician control of decisions and explicit recommendations may negatively impact the patient’s ability to manage behaviors [32]. Moreover, our results suggest that there is a risk that the degree of personalization in the form of protecting the patient can result in benevolent bias. This is evident in our participants’ recognition that their patients often initiate the conversation around risk. Patients desire direct conversations about diet, physical activity, and weight management in their overall survivorship care [11]. Yet, it may be that oncologists’ benevolent bias prevents these discussions from taking place and potentially diminishes patients’ beliefs about the importance of addressing health risks.

A patient’s mental representation of a health risk and the perceived value of engaging in risk management behaviors can be impacted by the quality of communication their clinician provides [37]. For example, risk communication scholars have demonstrated that clear and detailed communication about individual risk is more effective than relaying a relative risk [38, 39]. This approach involves providing clear action plans, explaining the potential causes and consequences of their risk, highlighting the benefits of actions, and reassuring patients of their ability to manage the threat [37]. Comprehensive communication can help patients better understand their risk [40] and positively influence patient engagement in risk management strategies [18, 41]. In the context of cancer survivorship care, explicit discussions of risk behaviors emphasizing the significance of risk reduction and offering encouragement may enhance survivors’ self-efficacy and motivation and may improve patient adherence to lifestyle risk behavior management [17–19, 42].

Despite participants reporting that they occasionally discuss general lifestyle behaviors with cancer survivors, their tendency to avoid direct conversations about individual risk behaviors appears influenced by a well-intentioned, benevolent bias. This avoidance can lead to inadequate risk management by clinicians and cause patients to undervalue the importance of specific risk factors, potentially resulting in worse health outcomes. To improve long-term health for cancer survivors, oncologists may need support to be more fully engaged in specific risk management for cancer survivors. The behavior change techniques used to address poor diet, smoking, and physical inactivity are effectively the same [43], and there may be value in work addressing all lifestyle risk behaviors together to ensure patients meet all the appropriate guidelines and recommendations for cancer survivors [2, 5, 26]. Integrating clinicians with a program that addresses multiple risk behaviors simultaneously could provide clinicians with an avenue to initiate a conversation about all three risks with their cancer survivors and help them overcome their benevolent bias. This type of effort can help clinicians deliver holistic risk behavior management to cancer survivors.

This study has limitations. Only one midwestern academic healthcare system was involved. Additionally, the interview’s objective was to gain insight into how to assist oncologists in implementing a telehealth risk behavior program for cancer survivors; as such, nurses, social workers, internal medicine clinicians, and others often involved in a cancer survivor’s care were not interviewed. Lastly, we did not ask participants to describe their discussions around alcohol use; although, some brought it up organically as a risk factor that they might discuss.

## Conclusion

This study explored oncologists’ reported communication strategies and attitudes on discussing risk behaviors with cancer survivors and highlights a delicate balance between patient-centered communication and benevolent bias. Many participants recognized that they may not always discuss specific risks with patients, like weight, or frame risk behaviors more generally to avoid damaging their relationship and the patient’s self-image. While participants often practiced patient-centeredness by personalizing their conversation about risk according to the specific diagnosis, treatment, and patient needs, tailoring the conversation based on what they think a patient can handle can lead to conversation avoidance around sensitive risk topics, ultimately resulting in less personalized and indirect risk discussions. This well-intentioned approach may lead to inadequate risk management by the clinician and result in patients underestimating the importance of specific risk factors, potentially leading to poorer health outcomes.

Oncologists must recognize the line between patient-centered communication and their benevolent bias to enhance communication effectiveness and avoid unmanaged risk behaviors. These findings suggest that oncologists may need support to be more fully engaged in specific risk management conversations with cancer survivors. Risk management programs that address multiple risk behaviors simultaneously could provide clinicians with an avenue to initiate a conversation about all three risks with their cancer survivors. This type of effort may help clinicians deliver holistic risk behavior management to cancer survivors.

## Supplementary Information

Below is the link to the electronic supplementary material.

ESM 1 (DOCX 47.3 KB)

## Data Availability

No datasets were generated or analysed during the current study.
