# Supplementary material for: Speaking of survival: oncologists’ approaches to risk behavior conversations with cancer survivors
Source: J Cancer Surviv. Author manuscript; Available in PMC 2026 Oct 20. (PMC13676549; doi:10.1007/s11764-025-01798-1)
Supplement: Supplementary file 1 [file NIHMS2214050-supplement-Supple_Material.docx]

**Speaking of Survival: Oncologists’ Approaches to Risk Behavior Conversations with Cancer Survivors**

Savanna Kerstiens^1^ MA, Courtney Lynam Scherr^3^ Ph.D., Maia Jacobs^1^ Ph.D., Allison J. Carroll^2^ Ph.D., Elyse Renee Daly^2^ BA, Monisola Jayeoba^1^ MS, Siobhan M. Phillips^3^ Ph.D., MPH, Brian Hitsman^3^ Ph.D., Sofia F. Garcia^3^ Ph.D., & Bonnie Spring^3^ PhD

^1^Northwestern University, School of Communication, Evanston, IL.

^2^Northwestern University, Feinberg School of Medicine, Chicago, IL.

^3^Robert H. Lurie Comprehensive Cancer Center of Northwestern University, Chicago, IL.

**Corresponding Author:** Correspondence concerning this article should be addressed to Courtney Scherr, Department of Communication Studies, Frances Searle Building, 2240 Campus Drive, Evanston, IL 60208. Email: [courtney.scherr@northwestern.edu](mailto:skerstiens3@u.northwestern.edu)

**Clinician Semi-Structured Interview Guide**

**Welcome and overview of the Interview:** Thank you for agreeing to participate in this study today. We sincerely appreciate your time and your expertise. The goal of this interview is to hear what you think about a new telehealth health promotion intervention study we will soon launch and how you’d like to be informed of your patients’ participation and progress. The most important thing to keep in mind is that there is not a right answer to any of our questions. We are genuinely interested in your thoughts, experience and expertise. Importantly, we want to know how we can support you if one or more of your patients decides to participate in our study. Before we get started, do you have any questions for me?

To begin, we would like to learn whether and how you currently address health risk behaviors in clinical practice.

**INTRO: Addressing Health Risk Behaviors in Clinical Practice.**

1) We recognize that you have limited time with each patient, and many important things to accomplish during a visit. Where does talking about health promotion behaviors like quitting smoking, becoming more physically active, and managing weight fall in the list of priorities for a patient encounter?

*Note: If clinician does not talk about these risk behaviors or indicates they do not have time, skip to question 2.*

a) How do you usually handle a patient who smokes, is physically inactive or overweight?
b) We know that patients often engage in more than one health risk behavior: for instance, they may smoke, be inactive and overweight. How do you talk about risk behavior bundles with these patients? What kinds of strategies do you use when you talk about changing multiple (bundled) risk behaviors?

2) Do patients ever bring up these health behaviors?
   a) How do those conversations go?
   b) What referrals, if any, do you make?

**ORIENTATION TO STELLAR: Reaction to Program and Trial**

*Now I’m going to explain a little bit about our study. The STELLAR study evaluates a yearlong health promotion intervention to treat the three most common risk behaviors that impair cancer survivors’ quality of life and increase their risk of new cancers and cardiovascular disease. These 3 behavioral risk factors are smoking, physical inactivity, and obesity. Our team is experienced in delivering evidence-based treatments for each of these risk behaviors that have been shown to be effective in multiple high-quality RCTs. The aim of the STELLAR research study is to evaluate a way of bundling these treatments into a single service to make them scalable: accessible to all cancer survivors, fully reimbursable & without workflow interference. Treatment is offered via telehealth by Health Promotionists. Health promotionists are expert health coaches who are well-trained in using the mobile-health tools (app, fitbit, wifi scale) that help patients track their behaviors. They provide support and hold patients accountable for their behavior and progress. Health Promotionists in this study are supervised by licensed clinical health psychologists to deliver the evidence-based treatment for cancer risk behaviors that many oncologists would like their patients to have but don’t have time or training to deliver.*

3) What are your initial thoughts about STELLAR?
   a) What concerns do you have about STELLAR?
      i) Do you anticipate any challenges?
   b) What do you think about the idea of offering these health promotion services remotely instead of in-person in a clinical setting?
   c) How do you feel about the health promotionist’s role?
      i) How can they best support the rest of the clinical team?
4) What do you imagine your role would be in the STELLAR program?
   a) What are your thoughts about recommending the STELLAR program to your patients?
      i) What might encourage you to recommend the STELLAR program?
      ii) What might prevent you from recommending the STELLAR program?
   b) If one of your patients asked you about the STELLAR program, how might you respond?
   c) What additional training do you think you might need from us in order to feel comfortable discussing STELLAR with your patients?

**PATIENT PARTICIPATION:** **Interest in knowing patient participation and progress**

Now we would like you to think more about the possibility that one of your patients enrolls in the STELLAR intervention.

5) If one or more of your patients **enrolled** in the STELLAR program, do you want to be notified about their enrollment?
   a) [If YES] How would you like to be notified of their enrollment?
   b) [If NO] Why not?
   c) Would you like anyone else on your care team to receive patient enrollment information?
6) If one or more of your patients were enrolled in the STELLAR program, how do you want to be notified of their progress?
   a) When would you like to be notified of their progress?
   b) If you received information about their progress at a time when you were not going to see them in clinic, would you look at the information? Would you message them about their progress?
7) Although we are unsure if it is feasible at this moment, would you ever have any interest in being able to view sensor data for your patients (e.g., wifi scale, fitbit)?

For your patients **who are enrolled** in STELLAR, we will be capturing their progress over time, and would like to know if any of this information would be useful to you.

First, I would like to show you an example of an Inbasket message that we are thinking about sharing with patients’ oncologists. Here is the first example [SHOW EXAMPLE INBASKET MESSAGE]

8) Looking at this message, what are your initial thoughts?
   a) Can you tell me what this message means to you? In other words, how do you interpret this information?
      i) How do you interpret the terms “Achieving” and “Not meeting goals”?
   b) What information in this message appeals to you or do you find useful?
      i) What aspects of this message must we keep in future versions?
   c) What information is unnecessary or unhelpful?
   d) What information is missing?
   e) How might you use this information in patient care?
9) Would a script from the STELLAR team that outlines how to discuss the patient’s progress with him or her be helpful? [refer back to prototype – ask if they want more extended talking points]]
   a) What are your initial thoughts? What would you change?
10) Who else on the care team do you think should see these kinds of Inbasket messages?

Next, I am going to show you a set of visuals that depict patient progress over time. and could be integrated into a dashboard in the electronic medical record. [SHOW EXAMPLE GRAPHS]

11) Looking at these graphs, what are your initial thoughts?
    a) Can you tell me what each graph means to you? In other words, how do you interpret the information that the graphs are showing?
       i) Does it look to you like the patient is meeting or missing their goals? [probe as needed to understand their interpretation of the graphs]
          (1) What indicates to you that they are meeting their goals?
          (2) What indicates to you that they are missing their goals?
    b) What is appealing or useful about these graphs?
    c) What is confusing about these graphs?
    d) Do you imagine looking at these visuals during the clinical encounter?
       i) If yes, how do you imagine using them, or what would you do after looking at them?
       ii) If no, would you look at them at another time?
    e) Who else on the care team do you think would benefit from seeing these graphs?
12) As you may know, it is sometimes helpful for patients to work on smaller goals – like smoking only 3 cigarettes per day – as they work toward a final goal – like zero cigarettes per day. Do you think it is more helpful to see patients progress in a graph mapped onto their smaller personal goals or onto the final goal?
13) What period do you think is helpful to see? For example, we can show patients’ progress over the past 4 weeks, 3 months, 6 months, etc.?
14) I would like you to think about where you spend the most time in the EHR when you are interacting with a patient. Where do you most often look? (e.g., flowsheet, snapshot, other)
    a) Where would you prefer to see graphs like the ones you just saw?
    b) Would you be likely to access them if you were directed to them from an In-basket message?
15) How often would you like to see updates on patients’ progress? Is there a time when you are most likely to look at this information (e.g., just before a patient encounter, morning before, evening before)?
16) What do you think will be important for us to do in the STELLAR program to make it sustainable within comprehensive cancer clinical care?
17) Before we move to our demographic questions, is there anything else that you think is important for us to know as we continue to develop our STELLAR intervention and move it to clinical implementation?

**SOCIODEMOGRAPHICS**

Thank you for answering the questions so far. This last set of questions asks about you and your practice.

1. What is your age? ______
2. With which gender identity do you most identify?
   a. Woman
   b. Man
   c. Transgender man/trans man
   d. Transgender woman/trans woman
   e. Genderqueer
   f. Don’t know
   g. Prefer not to answer
   h. Other (please specify):
3. How would you describe yourself? (You can select more than one)
   a. American Indian or Alaska Native
   b. Asian
   c. Black or African American
   d. Native Hawaiian or Other Pacific Islander
   e. White
   f. Other
4. Are you of Hispanic, Latino, or of Spanish origin?
   a. Yes
   b. No
5. Do you speak any other language(s) in clinic?
   a. Yes
      i. What other language(s) do you speak? _______________
   b. No
6. How many years have you practiced medicine? _________
7. What is your clinical specialty?_______________
8. Approximately what percentage of your patients are in survivorship [i.e., post- primary treatment, post- treatment with curative intent?___________
9. Practice location
   a. Downtown Chicago
   b. Northern Region (Lake Forest, Glenview, Grayslake)
   c. Western Region (Geneva, Warrenville, Winfield, DeKalb, Sandwich)
   d. Valley West
   e. Northwest Region (McHenry, Huntley)
   f. South Region (Palos Heights, Orland Park)
   g. Other (please specify)
10. Can you tell me a best estimate of the approximate percent of your patients who have each insurance type?
    a. % patients Medicaid ________
    b. % patients Medicare ________
    c. % patients private insurance ________
    d. % patients self-pay or uninsured ________
    e. I can’t say

Thank you so much for your time today, we really appreciate your insight and interest in our project. Do you have any other questions for me?
